# Review of Ocular Manifestations of Joubert Syndrome

**DOI:** 10.3390/genes9120605

**Published:** 2018-12-04

**Authors:** Stephanie F. Wang, Tia J. Kowal, Ke Ning, Euna B. Koo, Albert Y. Wu, Vinit B. Mahajan, Yang Sun

**Affiliations:** 1 State University of New York Downstate Medical Center, Brooklyn, NY 11203, USA; stephanie.wang@downstate.edu; 2 Department of Ophthalmology, Stanford University School of Medicine, 1651 Page Mill Road, Palo Alto, CA 94305, USA; tjkowal@stanford.edu (T.J.K.); vikaning@stanford.edu (K.N.); eunakoo@stanford.edu (E.B.K.); awu1@stanford.edu (A.Y.W.); vinit.mahajan@stanford.edu (V.B.M.); 3 Palo Alto Veterans Administration, Palo Alto, CA 94304, USA

**Keywords:** Joubert syndrome, primary cilia

## Abstract

Joubert syndrome is a group of rare disorders that stem from defects in a sensory organelle, the primary cilia. Affected patients often present with disorders involving multiple organ systems, including the brain, eyes, and kidneys. Common symptoms include breathing abnormalities, mental developmental delays, loss of voluntary muscle coordination, and abnormal eye movements, with a diagnostic “molar tooth” sign observed by magnetic resonance imaging (MRI) of the midbrain. We reviewed the ocular phenotypes that can be found in patients with Joubert syndrome. Ocular motor apraxia is the most frequent (80% of patients), followed by strabismus (74%) and nystagmus (72%). A minority of patients also present with ptosis (43%), chorioretinal coloboma (30%), and optic nerve atrophy (22%). Although mutations in 34 genes have been found to be associated with Joubert syndrome, retinal degeneration has been reported in only 38% of patients. Mutations in *AHI1* and *CEP290*, genes critical to primary cilia function, have been linked to retinal degeneration. In conclusion, Joubert syndrome is a rare pleiotropic group of disorders with variable ocular presentations.

## 1. Introduction

**Figure 1:**
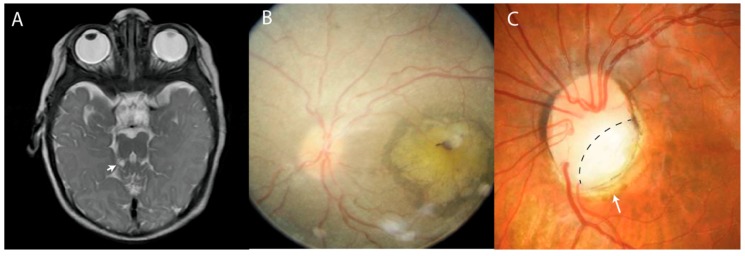
Representative clinical ocular presentations of Joubert Syndrome. (**A**) Magnetic resonance imaging (MRI) T2-weighted image of the “Molar Tooth Sign” (white arrow). (**B**) Retinal dystrophy with maculopathy. (**C**) Coloboma of the optic disc, marked with a dashed line.

Joubert Syndrome (JS) is a rare, autosomal recessive disorder with findings of episodic hyperpnea, global developmental delays, ataxia, and abnormal eye movements first described by Marie Joubert in 1969 [1,2,3]. It is accompanied by a congenital malformation of the brainstem and cerebellar vermis that composes the pathognomonic finding of a “molar tooth sign” (MTS) on magnetic resonance imaging (MRI) of the brain (Figure 1A). Abnormal features that constitute MTS include cerebellar vermis hypoplasia, deepened interpeduncular fossa, and thickened and elongated superior cerebellar peduncles. There are many subtypes of JS and JS-related disorders where MTS is found that involve the brain, eyes, kidneys, liver, and limbs [4,5].

The heterogeneous clinical findings of this genetic disorder are due to defects in primary cilia, a subcellular organelle that is a component of all vertebrate cells. The primary cilium is a microtubule-based organelle that protrudes from the cell surface and acts as an antenna to sense the extracellular environment. Its pathogenesis places JS within an expanding spectrum of diseases termed ciliopathies [6,7]. Commonly found features in JS include ophthalmic and oculomotor abnormalities, which can be useful in establishing a diagnosis [2]. The diverse abnormal eye movements include strabismus, nystagmus, oculomotor apraxia, and vertical gaze palsy. Several case reports have described retinal abnormalities (Figure 1A–C); among them are retinitis pigmentosa (RP), retinal dystrophy, chorioretinal colobomas, and retinal blindness [8,9,10,11,12,13]. This review will detail the ophthalmologic findings of JS. 

## 2. Epidemiology

Because of its rarity, there is little epidemiological data on JS, but it is reported to have a prevalence of between 1/80,000 and 1/100,000 live births [5,14,15]. This may be an underestimate due to its heterogeneous presentation [4]. 

The prevalence is higher in certain ethnic groups. These include French Canadians, in whom multiple pathogenic mutations in the *C5ORF42* gene (also known as CPLANE1) have been reported since Joubert et al. described the initial cases in 1969 [16]. Pathogenic mutations were identified in 33/35 (94%) French Canadian families tested [17] and included the following genes: *CPLANE1*, *CC2D2A*, *NPHP1*, *TMEM231*, *CEP290*, *TMEM67*, *TCTN1*, *OFD1*, *B9D1*, *C2CD3*, and *CEP104*. Many French Canadian patients were found to be compound heterozygous for pathogenic mutations in the genes *CPLANE1*, *CC2D2A*, *TMEM231*, and *NPHP1* [16,17,18].

The incidence of JS is also higher in Ashkenazi Jews and Hutterites [19,20]. The prevalence of JS is as high as 1/34,000–1/40,000 in Ashkenazi Jews because of the pathogenic mutation p.R73L in the *TMEM216* gene, as well as a higher carrier frequency of 1:90–1:100 [19]. In the Canadian Hutterite population, the carrier frequency of a pathogenic mutation, the founder mutation p.R18X in the *TMEM237* gene, is even greater at 1:17, which increases the prevalence of JS to approximately 1/1150 [20]. Additionally, two different Schmiedeleut Hutterite families share the same pathogenic mutation, c.363_364delTA in *CSPP1*, which indicates a separate founder variant [21]. An investigation of Japanese families with JS found that 22% had pathogenic mutations in *CEP290*, with c.6012-12T>A found on nine out of 12 disease alleles; 26% of families had pathogenic mutations in *TMEM67* [22]. Thus, targeted genetic analysis should be considered for these groups [4], as well as for the Dutch (founder mutation p.Arg2904Ter in *CPLANE1*) [14], French Canadian (variants in *CPLANE1*, *CC2D2A*, *NPHP1*, and *TMEM231*) [17], and Japanese (c.6012-12T>A mutation in *CEP290*) [22].

## 3. Clinical Classifications

The classic form of JS consists of a distinct triad of clinical signs: hypotonia, developmental delays, and pathognomonic midbrain–hindbrain malformation (MTS) on MRI [4]. Breathing abnormalities (episodic tachypnea or apnea) and/or unusual eye movements may be associated with the triad. The breathing abnormalities usually improve as the patient gets older, but truncal ataxia develops or becomes more apparent, and the attainment of age-appropriate motor skills is delayed [4]. Intellectual capacity varies and can range from normal to severely disabled. Additional abnormalities that may be associated with JS include renal disease, occipital encephalocele, polydactyly, ocular colobomas, retinal dystrophy, hepatic fibrosis, oral hamartomas, and endocrine disorders [4]. 

Due to its heterogeneous, multi-organ pathology, JS is classified into eight phenotypic subgroups based on the main system(s) involved: pure JS, JS with renal abnormalities, JS with ocular abnormalities, JS with oculo-renal abnormalities, JS with orofaciodigital abnormalities, JS with hepatic defects, JS with acrocallosal features, and JS with Jeune asphyxiating thoracic dystrophy [4,5]. Just as the authors who originally designated these classification subtypes, we suggest that due to the heterogeneity of presentation of JS, these classifications should not be considered as definitive. 

In the classic form of JS, patients exhibit muscle weakness/ataxia, developmental delay, and MTS along with variable degrees of abnormal breathing, eye movements, and cognitive ability. Renal, liver, and retinal disorders are absent [4]. Mutation in several genes has been found in these classic cases [4]. Patients with JS with ocular defects (JS-O) suffer from retinal dystrophy that varies in progression and severity in addition to the abnormalities of the classic form; *AHI1* mutations are frequent in this subgroup [4].

Patients with JS with renal disease (JS-R) exhibit the features of a kidney disorder that is predominantly juvenile nephronophthisis (NPH), without defects in the retina; mutations of *NPHP1* and *RPGRIP1L* are associated with this phenotype. Patients with JS with oculorenal defects (JS-OR) suffer from both retinal dystrophy and renal defects; *CEP290* mutations account for approximately 50% of these presentations [4]. In JS with hepatic defects (JS-H), individuals also suffer from congenital hepatic fibrosis; ocular and renal defects may occur but are not required for inclusion in this subtype. Mutations in *TMEM67* account for more than 70% of these cases [23,24]. In JS with orofaciodigital defects (JS-OFD), patients present with polydactyly, a bifid or lobulated tongue due to soft-tissue nodules or multiple hamartomas, and multiple oral frenulae. Hypothalamic hamartoma may occur, and the pituitary gland may be absent. This presentation is associated with mutations in the *TMEM216* gene [5]. In JS with acrocallosal features (JS-AC), there is complete or partial absence of the corpus callosum [25]. One series reported corpus callosal abnormalities of varying degrees of severity in 16 of 20 cases with JS [26]. Callosal abnormalities are also relatively frequent in biallelic *KIF7* pathogenic variants of JS [27], suggesting overlap with acrocallosal syndrome in which polydactyly and hydrocephalus are also seen.

Several cases of JS with Jeune asphyxiating thoracic dystrophy (JS-JATD) have been observed [28,29]. Symptoms of JATD, a rare autosomal recessive disorder, and of the related Mainzer–Saldino syndrome, include a long, narrow thorax; short stature with undersized limbs; polydactyly; and renal cystic dysplasia. The number of genes associated with JS continues to increase, along with greater recognition of the importance of cilia function in the development of multiple organ systems.

## 4. Genetics

**Figure 2:**
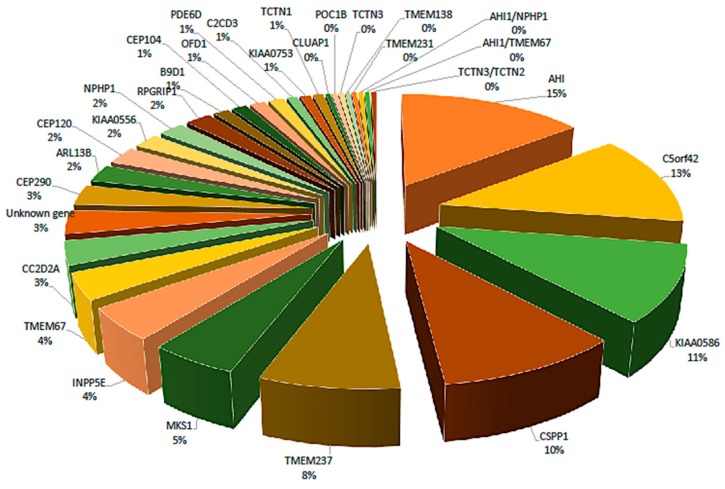
Proportion of mutations in different genes based on 245 patients with Joubert syndrome. Mutations were observed in 28 genes, but approximately half of the mutations were found in only four genes: *AHI* (15%), *C5orf42* (13%), *KIAA0586* (11%), *CSPP1* (10%).

Joubert Syndrome is categorized among the ciliopathies because its pathogenic genes generate proteins that are important in the function of primary cilia [30]. Twenty-eight identified genes are known to be involved in the pathogenesis of JS; most alleles are autosomal recessive. The most common genes are *AHI1*, *CC2D2A*, *CEP290*, *CPLANE1*, *CSPP1*, *INPP5E*, *KIAA0586*, *MKS1*, *NPHP1*, *RPGRIP1L*, *TMEM67*, and *TMEM216*. Less common genes include *ARL13B*, *B9D1*, *C2CD3*, *CEP104*, *CEP120*, *KIAA0556*, *OFD1*, *PDE6D*, *POC1B*, *TCTN1*, *TCTN3*, *TMEM138*, *TMEM231*, and *TMEM237.* These genes are important for establishing the clinical diagnosis of JS. A genetic diagnosis can be made in over 60% of individuals through identification of homozygous mutations in one of the 27 autosomal recessive genes or a heterozygous variant in the X-linked gene (*OFD1*) (Figure 2) [4]. 

Genetic testing that utilizes multigene panels for targeted examination of genes reported to be related to JS can therefore be applied. Alternatively, whole genome sequencing, which is more comprehensive, will aid in correctly diagnosing patients with JS. It is recommended that genetic testing begin with a multigene panel, and that genome sequencing be used if the multigene approach identifies no known variations [31]. Sequence analysis, deletion/duplication analysis, and non-sequencing-based tests are often utilized in the multigene panel [4]. Genetic testing for mutations causing JS can aid clinicians to develop appropriate management plans based on the expected phenotypes associated with these genes.

In patients with classic JS or JS with ocular involvement, the genes most commonly mutated are *AHI1*, *INPP5E*, *ARL13B*, and *CC2D2A* [6,32]. The AHI1 protein has been found to maintain appropriate vesicular trafficking to cilia; this trafficking is necessary for the normal function of photoreceptor outer segments. Indeed, in mice, the retina deteriorates in the absence of *AHI1* [33]. Interestingly, in a cohort of 100 JS patients, no retinal degeneration was observed in patients with *TMEM67*, *C5orf42*, or *KIAA0586* mutations, which are among the most common identified with JS [31]. However, another study using a similar set of patients from the same clinical study identified patients with thinning of the retinal nerve fiber and optic pallor associated with mutation in *KIAA0568* [34]. Additionally, a study of a cohort of Northern Europeans identified retinal dysplasia associated with mutation in *TMEM67* [14]. Despite the heterogeneity in the genes involved, several ophthalmic phenotypes (Table 1) are common, including retinal dystrophies and oculomotor defects. 

## 5. Ophthalmic Phenotypes/Presentation

**Figure 3:**
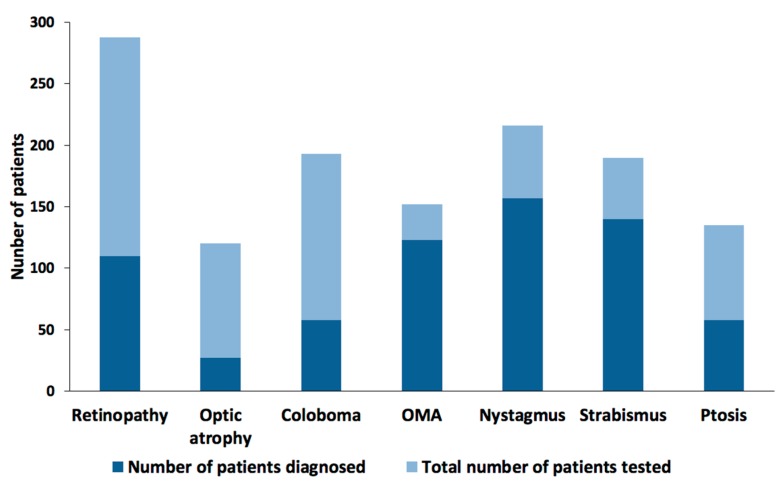
Frequency of phenotype observed based on the number of patients analyzed. OMA: Oculomotor Apraxia.

**Figure 4:**
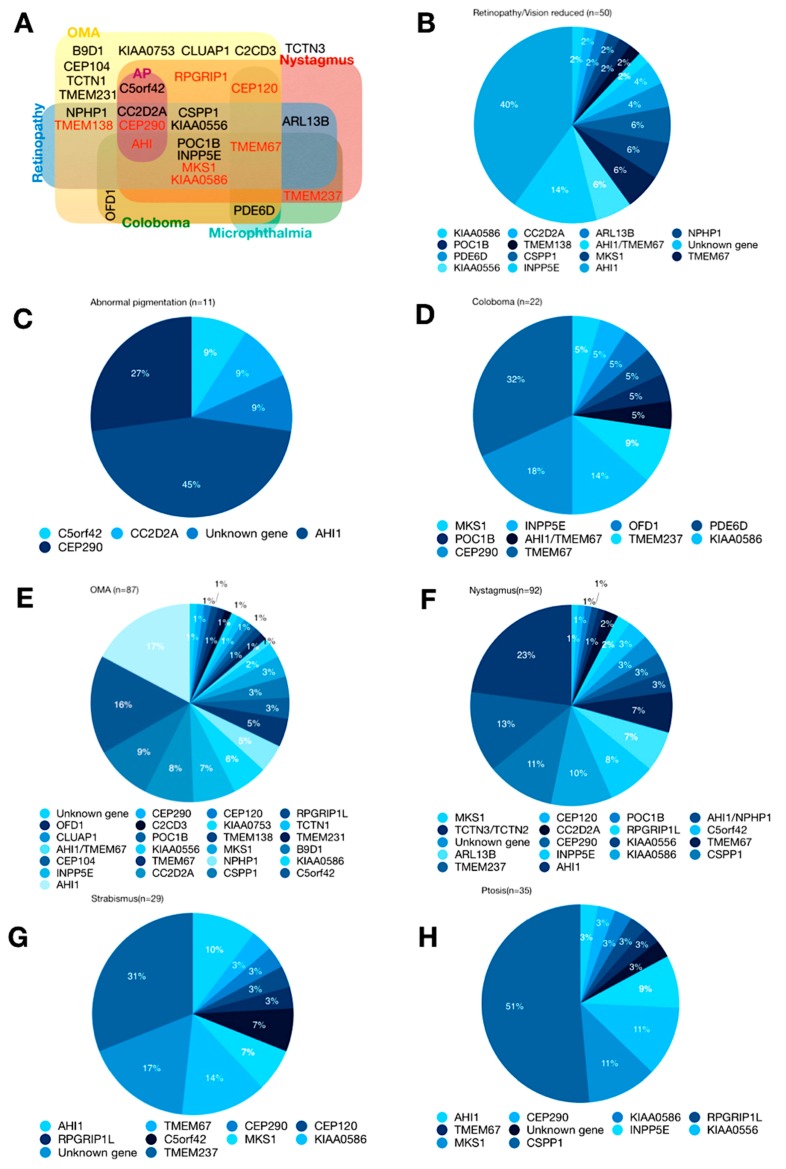
(**A**) Genetic overlap within the eye phenotypes of Joubert Syndrome. Most of the genes are associated with OMA and nystagmus. AP = Abnormal pigmentation. (**B**–**H**) Pie charts showing the prevalence of genetic causes in the entire group of 254 patients diagnosed with different eye phenotypes. Genes are listed from left to right in order of increasing prevalence.

We surveyed 52 publications examining a total of 325 patients that have been diagnosed as having ocular phenotypes. These studies did not consistently test for the same ocular phenotypes (Figure 3). Discrepancies in the testing performed for each patient may be due to patient availability or ability to cooperate in testing. These discrepancies create a challenge for clinicians and researchers alike as the diagnosis and reported prevalence of the disease may be variable or underestimated. Commonly diagnosed phenotypes include retinal dystrophy, abnormal retinal pigmentation, ocular colobomas, oculomotor apraxia, nystagmus, strabismus, and ptosis, and are described in more detail below. Although in several cases one gene may correspond to only one phenotype, in general the phenotypes associated with each gene overlap (Table 1 and Figure 4A). 

## 6. Retinal Dystrophy

Retinal dystrophy is frequent in patients with JS (reported incidence ranges from 37 to 100%). Its presentation is so variable [9] that both “early-onset severe rod–cone dystrophy” and “late-onset cone–rod dystrophy” have been described. It is caused by progressive degeneration of retinal photoreceptor cells [2,4,5], which contain primary cilia (“connecting cilia”)—slender structures that connect the outer and inner segments [30]. Patients with retinal dystrophy have a higher incidence of multicystic renal disease and a higher mortality rate than JS patients without retinal dystrophy [34].

Retinal dystrophy can commonly be detected by autofluorescence photos of the retina [35]. Optical coherence tomography (OCT) may provide another diagnostic technique with which to investigate retinal ciliopathies [36]. In one patient with JS this modality revealed blurred external retinal layers in the macula center, “salt-and-pepper” fundus, inner segment–outer segment junction (IS/OS line) discontinuity, and a lack of external limiting membrane [36]. 

The gene most commonly observed to cause retinal dystrophy is *AHI1*, followed by *INPP5E*, but a total of 14 genes have been reported to be responsible for this phenotype (Figure 4B). The AHI1 protein, also called Jouberin [37,38,39,40], contains a highly conserved SH3 domain, suggesting a possible role as a signaling molecule. Indeed, it is known to interact with NPHP1 and Meckle, and may be involved in ciliogenesis or ciliary transport. *INPP5E* encodes an inositol polyphosphate-5-phosphatase [40,41], which also plays a well-documented role in ciliary signaling. The phenotypes of retinal dystrophy in JS also include RP, optic disc drusen, and Leber congenital amaurosis (LCA).

### 6.1. Retinitis Pigmentosa

Retinitis pigmentosa in JS typically manifests as spotting of the retinal pigment epithelium (RPE) and the thinning and reduction of retinal vessels. The primary cause of this defect originates in the connecting cilia of the retinal photoreceptor cells and the modified cilia of the outer segment [30]. 

Atypical RPE changes, which are unlike those of RP, have also been reported in JS. In one case the midperipheral fundus revealed uniformly distributed hypopigmented discrete lesions resembling those of retinitis albipunctata. Coarse intraretinal pigmentation along the retinal venules and general narrowing of arterioles were also observed [42]. 

Coats-like retinopathy has also been reported in one case of JS. The dilated and aneurysmal blood vessels of this exudative retinopathy were comparable to those of Coats disease, but more prominent in the inferior quadrants [43]. It was suggested that the Coats-like retinopathy may have been independent of JS, but Coats-like retinopathy has been previously described in association with LCA due to CEP290 mutation [44], which has also been linked with JS [45]. Similar retinal defects were found in three patients with Senior–Loken syndrome, which suggests that Coats-like retinopathy lies on the spectrum of retinal phenotypes present in ciliopathies [43]. Establishing the correct diagnosis is important for proper treatment as laser photocoagulation, cryotherapy, or vitreoretinal surgery can be performed in severe cases [43].

Other manifestations may include optic disc drusen, which seldom manifest in early childhood and which are infrequent congenital disc anomalies that are composed of hyaline substance and calcified deposits which accrue gradually over time. Only a few cases have been reported of JS with optic disc drusen; therefore, it is possible that the drusen occurred serendipitously in cases of JS and that the coexistence of JS and drusen should not be interpreted as an association until the pathophysiology of both is better understood [46]. Optic disc drusen should be suspected if optic disc swelling or elevation is observed without increased intracranial pressure [47,48]. Drusen are generally bilateral and occur with a prevalence of between 0.4 and 3.7% in a population that includes normal adults and children and of ~0.4% in the pediatric age group. In one case, orbital ultrasonography confirmed drusen in a 4-month-old patient with JS who presented with increased intracranial pressure. The procedure revealed suspicious hyperechoic lesions within both optic discs. The hyperechoic calcifications were more prominent with posterior shadowing compatible with drusen. In addition, it is intriguing that B-scan ultrasound and autofluorescence confirmed bilateral optic disc drusen in 2/10 subjects with JS; retinal dystrophy was also described in the first reported case of drusen in JS [11,49]. 

### 6.2. Leber Congenital Amaurosis

Leber congenital amaurosis is a severe retinal degeneration that causes extensive vision loss. Usually diagnosed soon after birth, it produces profound dysfunction of the two photoreceptor types, which leads to nystagmus, photophobia, reduced pupillary response, high hyperopia, and greatly reduced electroretinographic responses. Patients with LCA characteristically demonstrate a significantly greater than usual amount of eye poking and rubbing. This is described as Franceschetti’s oculo-digital sign and increases the risk of keratoconus in these patients [50,49]. Juvenile severe cone–rod or cone dystrophy (CORD, COD) and RP may be mistaken for LCA because of similar clinical features. 

Variants of 30 genes that have been identified as causing photoreceptor cell death and LCA are responsible for approximately 62% of cases. Although LCA is usually inherited in an autosomal recessive manner, a few cases presented with dominant inheritance. It can also appear as one component of a syndrome, as in JS [49,51,52,53,54,55,56]. One study of a consanguineous Iraqi family identified *POC1B*, a gene that is critical for ciliogenesis and for maintaining basal body and centrosome integrity, as a possible cause of JS with LCA and a polycystic kidney disease phenotype [57]. In one case of suspected JS with LCA, morphological analysis revealed an atrophic maculopathy and a significant decrease in photoreceptor cells. The disorganized retina contained almost no rods and only a few surviving cones in the perifoveal region. Pigmentation was decreased in most regions of the retina, and the RPE layer was thinned in peripheral regions. RPE65, an enzyme expressed in RPE cells, was detected in the RPE layer and in the outer segment, but the RPE layer did not contain lipofuscin granules, which indicates defective lysosomal degradation of phagocytosed photoreceptors. Centrin-2, a protein found in the basal bodies of cilia, was also absent.

### 6.3. Abnormal Pigmentation

Abnormal pigmentation was reported in 4.5% of the patients with JS in the publications reviewed (11/245). About 72% (8/11) of these patients carried an *AHI1* (5) or *CEP290* (3) mutation (Figure 4, Table 1); mutations were also detected in *C5orf42* (1) and *CC2D2A* (1), and the genotype of one patient was unknown (Figure 4C). The fundi of these patients demonstrated bone spicule or mottled pigmentary irregularities in the peripheral or mid-peripheral retina, or in the entire retina. In the clinic, pigmentary retinopathy with nystagmus or roving eye movements is frequently diagnosed as LCA. Interestingly, *CEP290* mutations also account for 20% of LCA (ref) [58], which may explain some of the overlap with other JS phenotypes. LCA, however, typically lacks a concomitant central nervous system disorder. 

## 7. Ocular Colobomas

Ocular colobomas are defects that arise from the fetal fissure failing to close normally, which leads to missing tissue in the retinal pigmented epithelium, the neurosensory retina, or the choroid [59]. These defects can be unilateral or bilateral, can affect the optic disc and/or choroid, and are most commonly localized to the posterior part of the eye [14,27,31,57,60,61,62,63,64,65,66,67]. Colobomas involving the iris have also been observed [68]. Colobomas comprise only a small subset of ocular defects across the JS subtypes, but have been reported in over 30% of JS patients with hepatic defects [69] and may also be associated with hepatic fibrosis [23]. Additionally, in a survey of 100 JS patients, chorioretinal coloboma was found to be associated with a decreased risk for retinal degeneration and increased risk for liver disease [31].

Differences in the incidence of coloboma between cohorts have also been observed. One study found that colobomas affected 19% of families with JS [70]. Another reported an incidence of 30% (28/99), with 20/28 involving the retina; those with the TMEM67 mutation had the highest incidence (80%) [35]. The consensus in the literature is that only ~3% of patients with JS have both coloboma and retinal degeneration [27,35,71].

Although the incidence of ocular colobomas is small, mutations in several genes have been identified in patients with this diagnosis (Figure 4D). These genes include *MSK1, INPP5E, ODF1, PDE6D, POC1B, AHI1/TMEM67, TMEM237, KIAA0586, CEP290*, and *TMEM67*. The gene associated with the greatest number of patients with ocular colobomas is *TMEM67* (Meckelin). This transmembrane protein plays an important role in ciliogenesis and signaling. 

### 7.1. Oculomotor Defects

#### 7.1.1. Oculomotor Apraxia

Common in JS is a distinct collection of neuro-ophthalmologic symptoms, which can include congenital ocular motor apraxia (OMA). OMA is the inability to voluntarily initiate saccades and patients are frequently observed thrusting their heads in the target direction of focus. Additionally observed symptoms include frequent cyclic deviations of the eyes, including periodic alternating gaze deviation, periodic alternating skew deviation, periodic alternating torsional deviation, and a lateral alternating skew deviation that produces an alternating hyperdeviation of the abducting eye. [8,36,43,57,60,63,67,72,73,74,75,76,77,78,79,80,81,82,83,84,85,86,87,88,89,90]. Wheel rolling torsional eye movements from extreme excyclodeviation to extreme incyclodeviation have also been observed in patients with periodic alternating skew deviation and paroxysmal skew deviation [91]. Brodsky et al. also suggested that a cyclical component underlies JS based on similarities between the abnormal breathing patterns and neuro-ophthalmologic features in a patient with JS who demonstrated episodic hyperpnea and apnea by Marie Joubert and intermittent nystagmus on eye movement recordings [91].

Oculomotor Apraxia is often noticed after infancy, which may be due to limited awareness of how it presents in the very young [92]. Most children with OMA present with propulsion of the head as a way to compensate for their inability to initiate saccades [9,12,93]. Horizontal head titubation has also been reported in infants and young children [84]. Oculomotor Apraxia is the phenotype with the largest number of mutated genes associated with it (Figure 4E). Twenty-five genes have been reported, including *ODF1, CLAUP1, AHI1/TMEM67, CEP104, INPP5E, AHI1, CEP290, C2CD3, POC1B, KIAA0556, TMEM67, CC2D2A, CEP120, KIAA07593, TMEM138, MSK1, NPHP1, CSPP1, RPGRIP1, TCTN1, TMEM321, B9D1, KIAA0586*, and *C5orf42*. Among them, *AHI1* has been found in the most cases of OMA. Despite the many identified genes, no abnormal gene has been found in numerous cases, and 17% of incidences are attributed to unknown genetic mutations. 

#### 7.1.2. Nystagmus and Strabismus

Abnormal eye movements, including primary position nystagmus, are a common feature in JS [11,14,43,57,65,67,72,73,77,79,84,85,86,87,90,94,95,96,97,98,99,100,101,102,103,104,105]. Fluxes in gaze holding, an early symptom, were described in a study of six patients with JS. Although the conjugate pendular nystagmus demonstrated before the age of 6 months is consistent with infantile nystagmus, the irregularities in conjugate eye movements of the subjects set apart the nystagmus found in JS from other forms of infantile nystagmus. The horizontal pendular nystagmus found in the patients was said to be similar to the periodic nystagmus in lesions of the uvula and nodulus [93]. Translation of the velocity signals transmitted by vestibular, pontine, and inferior olivary nuclei into the eye position is the basis for stable gaze holding [106].

Additionally, the see-saw nystagmus observed in this cohort was proposed to be associated with defects in the interstitial nucleus of Cajal (INC) located in the midbrain [93], since the INC is important in helping translate the vertical and torsional eye velocity signals transmitted by crossed and uncrossed inputs into position signals [106,107,108]. It was proposed that the loss of certain crossed inputs in JS patients may cause an imbalance of velocity inputs which leads to unstable gaze holding [93]. This is supported by studies of JS patients that point to decussation of the superior cerebellar peduncles and pyramidal tracts [10,109,110,111,112].

Nystagmus has been associated with mutations in the following genes: *MKS1, TCTN3/TCTN2, ARL13B, TMEM237, CEP120, CCD2D2A, CEP290, INPP5E, AHI1, POC1B, RPGRIP1L, KIAA0556, KIAA0586, AHI1/NPHP1, C5orf42, TMEM67, and CSPP1*. AHI1 gene mutations are the most frequently correlated with nystagmus (Figure 4F).

Strabismus is frequent in JS, as in other diseases associated with non-progressive cerebral or cerebellar abnormalities [113], and in one small series it was the most common neuro-ophthalmologic abnormality [113]. It can present as horizontal or vertical misalignment, esotropia or exotropia, fixed or alternating, but most often it presents as elevation of the abducting eye and depression of the adducting eye in lateral gaze [8,9,10,21,76,80,95,114,115]. In a study of neuro-ophthalmological findings in pediatric patients with chronic ataxia, it was found that diseases associated with non-progressive cerebral or cerebellar abnormalities like JS and related disorders were associated with high prevalence of strabismus. Four out of five of the patients with JS surveyed displayed neuro-ophthalmologic features, the most common feature being strabismus [113].

*TMEM237* gene mutation was most common in JS patients with strabismus (31% of cases). The protein product of this gene, a four-pass transmembrane protein that localizes to the ciliary transition zone, is known to be involved in ciliogenesis and Wnt signaling [20] (Figure 4G). Other genes identified with this phenotype include *AHI1, TMEM67, CEP290, CEOP120, RPGRIP1l, C5orf42, MKS1*, and *KIAA0586*.

### 7.2. Ptosis

Ptosis can be unilateral or bilateral and of varying severity in JS [65,77,97,99,101,102,114,115,116]. It occurred in 29% (28/98) of patients with JS assessed in one recent series [35]. It is found almost exclusively in children, but bilateral ptosis developed in one young adult in whom it was thought to be due either to progression of JS or to fibrocystic changes in the midbrain caused by ventriculoperitoneal shunting [117].

Mutation of the centrosome and spindle pole-associated factor-1 gene (*CSPP1*) is the most common mutation associated with individuals with JS and ptosis (Figure 4H). The CSPP1 protein localizes to the centrosome and is important in ciliogenesis [85]. One study of patients with *CSPP1*-related JS reported ptosis in 15/19 individuals with the biallelic truncating mutations [85]; another study found bilateral ptosis in 4/6 patients with CSPP1-related JS [86]. Additional genes implicated in JS-related ptosis include *AHI1, CEP290, KIAA586, RPGRIP1L, TMEM67, INPP5E, KIAA0556,* and *MSK1* (Figure 4), although one recent series did not find ptosis in subjects with mutations of *AHI1* or *KIAA0586* [35].

## 8. Conclusions

Joubert Syndrome is a group of complex multi-organ diseases that frequently present with ocular manifestations. The myriad disease presentations can range from mild ocular motility defects that improve with age to severe retinal degeneration that causes blindness at birth. The growing list of identified genetic mutations highlights the importance of the structure and function of primary cilia in the pathogenesis of JS. Development of animal models and targeted gene therapies will help advance personalized treatments for patients with this challenging group of diseases. 

## Figures and Tables

**Table 1:** Genes, proteins, and eye phenotypes in Joubert Syndrome.

JS-Related Gene	Protein	No. of pts	Eye Phenotypes									References (PMID)
			OMA	Nystagmus	Coloboma	Retinopathy (Vision Reduced)	Microphthalmia (Dysplasia)	Abnormal Pigmentation	Myopia/Hyperopia	Strabismus	Ptosis	
AHI1	Abelson helper integration site 1	36	+	+	+	+		+	+	+	+	28431631; 25920555; 26759440; 26541515; 16453322; 19461662; 19443711; 29987673; 21458016
ARL13B	ADP-ribosylation factor-like 13B	6		+		+						25138100; 29255182
B9D1	B9 domain containing 1	3	+									26477546; 24886560
C2CD3	C2 calcium dependent domain containing 3	2	+									26477546
C5orf42	CPLANE1, ciliogenesis and planar polarity effector 1	31	+	+				+				28431631; 26477546; 25920555; 25173907; 27866068; 24178751
CC2D2A	Coiled-coil and C2 domain containing 2A	8	+	+		+		+				26477546; 25920555; 27959436; 25173907; 29987673
CEP104	Centrosomal protein 104	3	+									26477546
CEP120	Centrosomal protein 120	6	+	+			+			+		27208211
CEP290	Centrosomal protein 290	7	+	+		+		+		+	+	26477546; 25920555; 19461662; 25818971; 24850569
CLUAP1	Clusterin associated protein 1	1	+									28679688
CSPP1	Centrosome and spindle pole associated protein 1	25	+	+		+					+	24360808; 24360807;
INPP5E	Inositol polyphosphate-5-phosphatase E	11	+	+	+	+			+		+	25920555; 25818971; 29052317; 20446224; 29987673
KIAA0556	KIAA0556	5	+	+		+					+	27245168; 26714646
KIAA0586	KIAA0586	26	+	+	+	+			+	+	+	26386247; 26386044; 26096313
KIAA0753	KIAA0753	2	+									28220259
MKS1	Meckel syndrome, type 1	12	+	+	+	+				+	+	27377014; 26490104; 24886560
NPHP1	Nephrocystin 1	5	+			+						26477546; 28347285; 23683649
OFD1	OFD1, centriole and centriolar satellite protein	3	+		+							26477546; 25920555; 28505061
PDE6D	Phosphodiesterase 6D	3			+	+	+					24166846
POC1B	POC1 centriolar protein B	1	+	+	+	+						25044745
RPGRIP1L	RPGR interacting protein 1	5	+	+						+	+	25920555; 19461662; 18565097
TCTN1	Tectonic family member 1	2	+									26477546; 26489806
TCTN3	Tectonic family member 3	1										25118024
TMEM138	Transmembrane protein 138	1	+			+				+		28102635
TMEM231	Transmembrane protein 231	1	+									25920555
TMEM237	Transmembrane protein 237	19		+	+					+		22152675
TMEM67	Transmembrane protein 67	9	+	+	+	+	+			+	+	28838911; 28431631; 26477546; 26166658; 25920555
AHI1/NPHP1		1		+								19443711
AHI1/TMEM67		1	+		+	+	+					25920555
TCTN3/TCTN2		1		+					+			25118024
Unknown gene	-	8	+	+		+		+		+	+	28838911; 19461662; 28018441

OMA = Oculomotor apraxia. Number of patients = 245.
